# A Deep Spatiotemporal Attention Network for Mild Cognitive Impairment Identification

**DOI:** 10.3389/fnagi.2022.925468

**Published:** 2022-07-18

**Authors:** Quan Feng, Yongjie Huang, Yun Long, Le Gao, Xin Gao

**Affiliations:** ^1^State Key Laboratory of Public Big Data, GuiZhou University, Guizhou, China; ^2^Faculty of Intelligent Manufacturing, Wuyi University, Jiangmen, China; ^3^Nanjing Huayin Medical Laboratory Co., Ltd., Nanjing, China; ^4^Department of PET/MR, Universal Medical Imaging Diagnostic Center, Shanghai, China

**Keywords:** functional brain network (FBN), mild cognitive impairment (MCI), graph convolution, attention, spatiotemporal features

## Abstract

Mild cognitive impairment (MCI) is a nervous system disease, and its clinical status can be used as an early warning of Alzheimer's disease (AD). Subtle and slow changes in brain structure between patients with MCI and normal controls (NCs) deprive them of effective diagnostic methods. Therefore, the identification of MCI is a challenging task. The current functional brain network (FBN) analysis to predict human brain tissue structure is a new method emerging in recent years, which provides sensitive and effective medical biomarkers for the diagnosis of neurological diseases. Therefore, to address this challenge, we propose a novel Deep Spatiotemporal Attention Network (DSTAN) framework for MCI recognition based on brain functional networks. Specifically, we first extract spatiotemporal features between brain functional signals and FBNs by designing a spatiotemporal convolution strategy (ST-CONV). Then, on this basis, we introduce a learned attention mechanism to further capture brain nodes strongly correlated with MCI. Finally, we fuse spatiotemporal features for MCI recognition. The entire network is trained in an end-to-end fashion. Extensive experiments show that our proposed method significantly outperforms current baselines and state-of-the-art methods, with a classification accuracy of 84.21%.

## 1. Introduction

Alzheimer's disease (AD) is an irreversible degenerative brain disease, and it is also one of the most common forms of dementia (Raju et al., 2020). AD usually occurs in the later years of human life. According to previous statistics released by the Global Health Organization, the global prevalence of AD reached a staggering 26.6 million in 2006, and this statistic will double every 20 years (Brookmeyer et al., 2007). In the future in 2046, 1.2% of the global population will be at risk of developing AD. Recent studies have shown that the prediction of mild cognitive impairment (MCI) is helpful for the early diagnosis of AD (Morris et al., 2001; Association, 2019). Because MCI is a clinical state between the normal population and patients with AD, it has a high probability of developing AD (Kang et al., 2020). In addition, individuals with features of MCI almost always have neuropathological features of AD (Morris et al., 2001). In medicine, MCI is a nervous system disease with the main symptoms being mild memory impairment and mild executive function impairment with additional visuospatial deficits (Gauthier et al., 2006). These symptoms are usually not life-threatening and can be detected by the patient or his or her family members. Current research suggests that MCI may not be a simple disease, but an early manifestation of AD disease (Ithapu et al., 2015). Therefore, MCI can often be regarded as an ideal clinical test subject for predicting AD disease. With the in-depth study of MCI, people can anticipate their risk for AD earlier and take preventive and therapeutic measures, such as taking oral medications to improve cognition (Roberson and Mucke, 2006) and changing their daily routine (Zubatiy et al., 2021). Since the variation between MCI and normal population is very subtle and slow (Association, 2019). Therefore, the prediction of MCI is a challenging task. After years of research, a large number of machine learning-based diagnostic methods have been developed for MCI identification (Li et al., 2017, 2019), which can be briefly classified into the following two types:

1. Based on traditional machine learning methods, which mainly use traditional machine learning techniques to model MCI data into a binary classification problem. For example, in Zhang et al. (2011), the authors capture and combine biomedical pattern features from different modalities with the help of multi-kernel support vector machines for predicting patients with MCI. In Liu et al. (2013b), authors adjusted the distribution of MCI-specific classes for MCI identification by a graph partitioning algorithm. In Liu et al. (2013a), the authors embedded high-dimensional neuro-imaging data into a low-dimensional space and exploited local sparse code gradients to test the data to further enhance the classification of MCI. Due to their strong reliance on prior-knowledge, these methods have strict dataset requirements, making it difficult to generalize in practical applications.
2. Deep learning based methods, mainly use the deep convolutional neural network (CNN) features to extract hidden features in neuroimaging data for MCI identification. For example, in Amoroso et al. (2017), authors designed a multiplexed neural network to model structural brain connectivity atrophy for the classification of MCI and normal controls (NC). In Yue et al. (2018), authors designed a 2DCNN framework to capture the most useful features in the gray matter of sMRI for MCI identification. In Puranik et al. (2018), the authors designed a deep 2DCNN framework and utilized the transfer method for AD, MCI, and NC classification. However, since these methods seldom consider the temporality of the existence of relevant data sets, their classification accuracy may be suboptimal.

**Figure 1:**
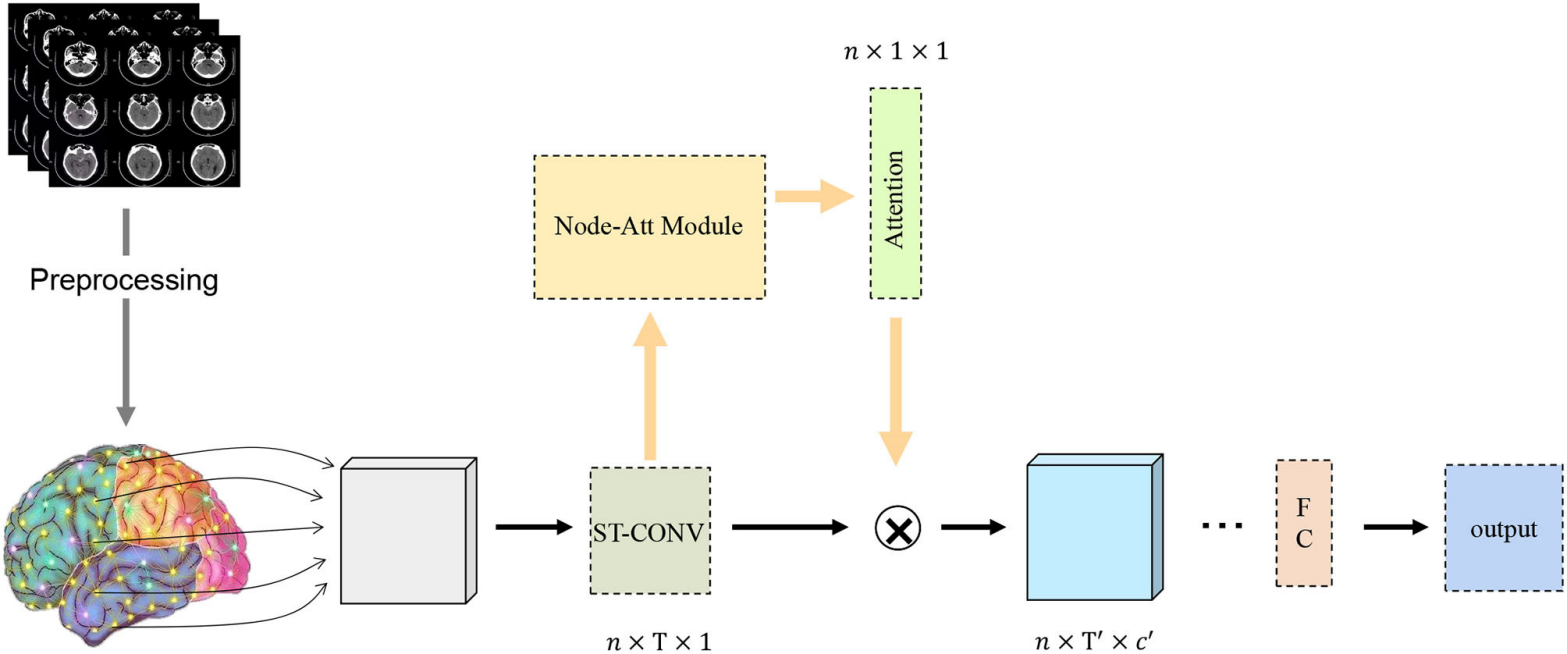
Deep Spatiotemporal Attention Network (DSTAN) structure illustration. Spatiotemporal convolution strategy (ST-CONV) represents spatiotemporal convolution, Node-ATT Module represents Attention module of brain functional Node, Attention represents brain node attention map and FC represents full connection layer. *n* represents the number of brain nodes, and T, T′ represents the number of time points of functional brain signals. *c*′ is the number of channels.

Despite the success of these methods, the identification of MCI is still a difficult problem. Excitingly, the functional brain network (FBN) has become an important method for modeling brain neural time courses, which provides an effective imaging biomarker for the diagnosis of MCI (Bray et al., 2021). A large number of medical experiments have found that the functional connections between brain regions, voxels and ROIs in FBN are highly correlated with some diseases such as nerves or MCI (Greicius, 2008; McKhann et al., 2011). Therefore, learning FBN based on time series correlation can provide more accurate and stable test results for MCI identification (Li et al., 2020b, 2021). In this article, FBN defines the nodes as brain regions, and the edge between these regions is determined by the relationship between their blood-oxygen-level dependent (BOLD) time series recorded by fMRI. In recent years, with the rise of deep graph convolutional networks, state-of-the-art performance has been achieved in their applications in different fields, such as social networks (Dowlagar and Mamidi, 2021; Liu et al., 2022), computer vision (Han et al., 2021; Zou and Tang, 2021), and gene prediction (Yu et al., 2021; Peng et al., 2022). Meanwhile, deep graph convolutional networks have also achieved satisfactory success in disease prediction tasks (Tang et al., 2021; Yu et al., 2021). Specifically, as shown in Figure 1, we first design a space-time convolution strategy (ST-CONV) to extract time-series features and structural features between brain functional signals and brain nodes. Then, we introduce an attention mechanism to further capture brain nodes that are more correlated with MCI. Finally, we fuse time series features and structural features (i.e., spatiotemporal features) for MCI identification. The whole network is trained in an end-to-end manner. Extensive experiments demonstrate that our proposed method is significantly competitive compared with the current baselines and the state-of-the-art methods. We summarize our main contributions as follows:

- A deep learning framework for MCI identification is proposed, which provides a new way for MCI identification.
- A new fusion mechanism is designed, which extracts the spatiotemporal features of brain functional signals and FBN, and applies its fusion to MCI identification.
- We used our DSTAN to distinguish MCI from NC and achieved a classification accuracy of 84.21%, which is superior to baseline and the most advanced methods.

The rest of this article is organized as follows: In Section 2, we introduce materials. In Section 3, we infer the DSTAN network. Section 4 reports the experimental results, and Section 5 discusses and looks forward to the full text.

## 2. Dataset

### 2.1. Data Acquisition

In this article, we use the same data set as Qiao et al. (2016). The data set consisted of 45 patients with MCI and 46 NC subjects, and these data were static Functional Magnetic Resonance Imaging (fMRI) images. At the same time, the data set can be obtained from the MCI database (https://www.nitrc.org/projects/modularbrain/), in which Table 1 is a summary of the demographic information of the subjects.

**Table 1:** Demographic information of subjects.

	**MCI**	**NC**
Gender (M/F)	25/20	14/32
Age (Mean ± SD)	74.13 ± 6.68	73.5 ± 3.50
MMSE (Mean ± SD)	27.71 ± 1.73	28.10 ± 1.35

### 2.2. Data Pre-Processing

In this section, we use fMRI images obtained by the standard echo planar imaging sequence function in the 3T scanner (TRIO, Siemens). During fMRI imaging, the parameters are set as follows: the voxel thickness is 2.97 × 2.97 × 3mm^3^, the number of slices is 45, acquisition matrix size is 74 × 74, and $\frac{\text{TR}}{\text{TE}}=\frac{3,000}{30}ms$ with 180 volumes. In addition, we use Statistical Parametric Mapping (SPM)^2^ and DPARSFA (version 2.2) for image pre-processing (Yan and Zang, 2010). In the pre-processing process, we discard the first 10 fMRI images of the subjects uniformly in order to prevent signal jitter. Then, we process the remaining fMRI images in the following steps: In step 1, we adopt a correction strategy for slice acquisition timing and head motion. In step 2, we remove the low and high-frequency artifacts in the corrected image and further regress out nuisance signals based on Friston et al. (1996). In step 3, we discard the time points with frame-wise displacement >0.5 to reduce the influence of micro-head movements on functional connectivity. On this basis, we divide the preprocessed BOLD time series signals into 90 ROIs according to the standard of automatic anatomical labeling (AAL) atlas. In step 3, we store these time series data of length 80 into a matrix of size *X* ∈ ℝ^80×90^.

## 3. Method

In this section, we design a DSTAN network, which captures spatiotemporal features by fusing temporal and spatial features of functional brain signals, and uses an attention mechanism to capture brain nodes related to MCI. Specifically, Section 3.1 formalizes the problem definition. Section 3.2 describes how to extract temporary and structural features in functional brain signals and functional brain networks. In Section 3.3, the attention mechanism is used to capture MCI related brain nodes. In Section 3.4, spatiotemporal features are fused. The objective function of DSTAN is defined in Section 3.5.

### 3.1. Problem Definition

Suppose the data set is $D={\left\{f{\left(x,t\right)}_{h},y_{h}\right\}}_{h=1}^{N}$, *N* denotes the number of samples, *f*(*x, t*)_*h*_ denotes the feature vector of the *h*-th sample, where *t*_*h*_ ∈ {*t*_1_, *t*_2_, …, *t*_T_}, *x*_*h*_ ∈ {*x*_1_, *x*_2_, …, *x*_*n*_}, and *y*_*h*_ ∈ {0, 1} is the corresponding label, and 0 and 1 represent “normal” and “MCI,” respectively. We assume that the FBN has *n* brain nodes corresponding to brain regions, G = {*V, E*}, where *V* denotes brain region and edge *E* denotes the functional connectivity between two brain regions. DSTAN networks have *L* convolution layers. The number of input and output channels in the *l*-th convolution layer is *c*_1_ and *c*_2_, respectively. For the *l*-th convolution layer, ${\text{f}}^{l}={\left\{f_{i}^{l}\left(x,t\right)\right\}}_{i=1}^{c_{1}}\in {\mathbb{R}}^{n\times \text{T}\times c_{1}}$ is the input of convolution, ${\hat{\text{f}}}^{l+1}={\left\{{\hat{f}}_{j}^{l+1}\left(x,t\right)\right\}}_{j=1}^{c_{2}}\in {\mathbb{R}}^{n\times \frac{\text{T}-w+1}{s}\times c_{2}}$ is the input of the brain node attention module, T denotes the number of time points of functional brain signals, *w* denotes the size of the convolution kernel, and *s* denotes the size of average pooling. The purpose of the DSTAN network design is to capture spatiotemporal features by fusing the spatial and temporal features of functional brain signals and to use the attention mechanism to focus on brain nodes related to MCI.

### 3.2. Spatiotemporal Convolution Strategy

**Figure 2:**
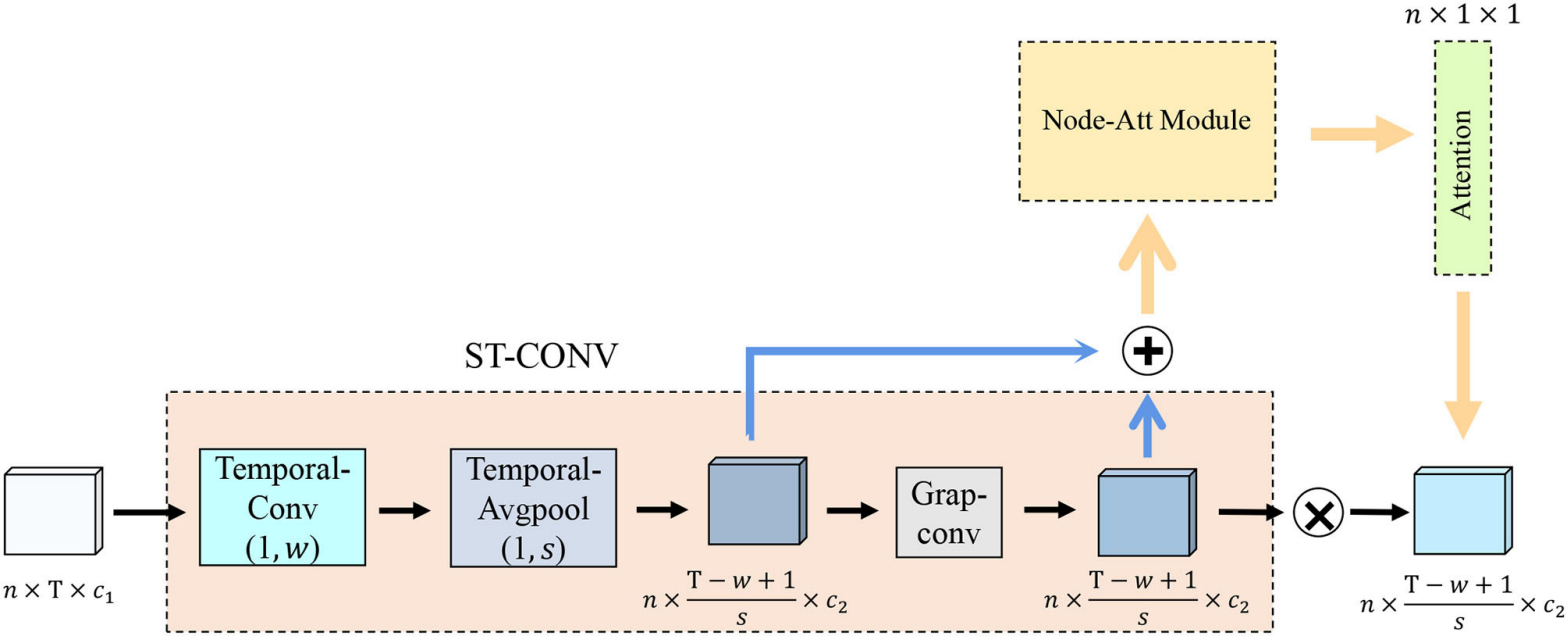
Spatiotemporal convolution strategy structure illustration. Temporal-Conv represents temporal convolution operation, Temporal-AvgPool represents temporal average pooling, Grap-Conv represents graph convolution operation, Node-Att Module represents the brain node attention module, and Attention represents the brain node attention map.

The transmission of functional brain signals is based on the underlying functional connections between brain regions (Huang et al., 2018), and they contain rich temporal information. Therefore, we extract node features (i.e., spatial features) and temporal features of functional signals from functional networks and time series, respectively. To this end, we design an ST-CONV strategy, as shown in Figure 2. We first perform convolution operation on the time series of input functional signals to extract its temporal features $f_{i}^{l}\left(x,t\right)$ as follows:

(1) $$\begin{array}{r} f_{j}^{l+1}\left(x,\frac{\text{T}-w+1}{s}\right)=\mathrm{tpool}\left(\sigma \left(\overset{c_{1}}{\underset{i=1}{\sum }}k_{j}\left(t,i\right)*f_{i}^{l}\left(x,t\right)\right)\right) \end{array}$$

where $f_{j}^{l+1}$ denotes the output, tpool(·) denotes temporal average pooling with a window size of (1, *s*), σ(·) denotes the activation function, and *k*_*j*_(*t, i*) denotes the convolution kernel with the size of (1, *w*) in the *i*-th channel. Then, we capture the spatial features of functional signals in the functional network through the graph convolution operation:

(2) $$\begin{array}{r} {\hat{f}}_{j}^{l+1}\left(x,t\right)=\sigma \left(\overset{c_{1}}{\underset{i=1}{\sum }}\left({\hat{D}}^{\frac{1}{2}}\hat{A}{\hat{D}}^{\frac{1}{2}}f_{j}^{l+1}\left(x,t\right)\text{W}\right)\right) \end{array}$$

where ${\hat{f}}_{j}^{l+1},j=1,2,\ldots ,c_{2}$ denotes the output of graph convolution, $\hat{A}=I+V$, $\hat{D}$ denotes ${\hat{D}}_{\text{nn}}=\underset{\text{m}}{\sum }{\hat{A}}_{\text{nm}}$, the rest elements are 0, *I* denotes identity matrix, W is the parameter corresponding to $f_{j}^{l+1}\left(x,t\right)$, and ${\text{f}}^{l+1}={\left\{f_{j}^{l+1}\left(x,t\right)\right\}}_{i=1}^{c_{2}}\in {\mathbb{R}}^{n\times \frac{\text{T}-w+1}{s}\times c_{2}}$ denotes the input.

### 3.3. Brain Node Attention Module

**Figure 3:**
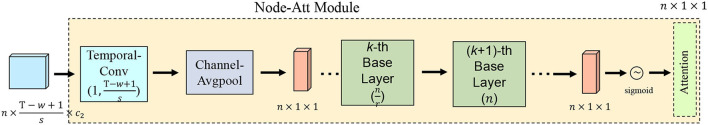
Illustration of attention mechanism. Temporal-Conv represents temporal convolution operation, Channel-AvgPool represents channel average pooling operation, and *r* represents the down-sampling rate.

There are a large number of brain nodes in the functional brain network, and the brain regions corresponding to different brain nodes reflect different diseases (Ries et al., 2008). In order to capture the features of brain nodes related to MCI, we introduce an attention mechanism, as shown in Figure 3. We first integrate the channel and time information of each brain node into a scalar, and solve the brain nodes related to MCI as follows:

(3) $$\begin{array}{r} {\text{M}}_{j}=\sigma \left({\text{h}}_{t}*{\hat{f}}_{j}^{l+1}\left(x,t\right)\right) \end{array}$$

where $\text{M}={\left\{{\text{M}}_{j}\right\}}_{j=1}^{c_{2}}\in {\mathbb{R}}^{n\times 1\times c_{2}}$ denotes the output of the *j*-th channel after convolution operation, h_*t*_ denotes the convolution kernel set with the size of $\left(1,\frac{\text{T}-w+1}{s}\right)$, and ${\hat{\text{f}}}^{l+1}={\left\{{\hat{f}}_{j}^{l+1}\left(x,t\right)\right\}}_{j=1}^{c_{2}}\in {\mathbb{R}}^{n\times \frac{T-w+1}{s}\times c_{2}}$ denotes the input of the brain node attention module.

Then, we maintain the feature invariance in the functional signal through the average down-sampling based on the channel dimension, and suppress the noise generated when collecting the functional signal to make it better for training, which can be expressed as:

(4) $$\begin{array}{r} C_{avg}\text{M}=\frac{\sum_{j=1}^{c_{2}}{\text{M}}_{j}}{c_{2}}\in {\mathbb{R}}^{n\times 1\times 1} \end{array}$$

where *C*_*avg*_**M** denotes the output.

In order to further capture the MCI-related brain nodes, we project the brain node features of *C*_*avg*_**M** into the MCI-related feature space. We design the *K*-layer base layer in the attention module and perform the following operations: 1) Perform down-sampling on the *k*-th base layer:

(5) $$\begin{array}{r} F^{k}=\text{relu}\left(fc\left({\text{C}}_{\text{avg}}\text{M},\frac{n}{r}\right)\right) \end{array}$$

where $F^{k}$ denotes the output of the *k*-th base layer, $\frac{n}{r}$ denotes the down-sampling rate, relu(·) denotes the activation function, and *fc*(·) is the same as the fully connected operation. 2) Perform up-sampling on the *k* + 1-th base layer:

(6) $$\begin{array}{r} F^{k+1}=\text{relu}\left(fc\left(F^{k},\frac{n}{r}\right)\right) \end{array}$$

where $F^{k+1}$ denotes the output of the (*k* + 1)-th base layer, and *n* denotes the up-sampling rate. In this way, we can further get the attention map of brain nodes as follows:

(7) $$\begin{array}{r} \text{Z}\left(x\right)=\mathrm{Sigmoid}\left(F^{k+1}\right) \end{array}$$

where **Z**(*x*) ∈ ℝ^*n* × 1 × 1^ denotes the brain node attention map. In the detailed process, the *k*-th base layer performs down-sampling from *n* brain nodes to $\frac{n}{r}$ brain nodes; the (*k* + 1)-th base layer performs up-sampling from $\frac{n}{r}$ brain nodes to *n* brain nodes. We use this nonlinear transformation to capture the dependency between brain nodes and MCI.

Finally, in order to further focus on the brain nodes with strong correlation, we multiply the brain node attention map with the functional signal:

(8) $$\begin{array}{r} {\tilde{\text{f}}}^{l+1}=\text{Z}\left(x\right)\times {\hat{\text{f}}}^{l+1} \end{array}$$

where ${\tilde{\text{f}}}^{l+1}$ denotes the output.

As discussed above, different brain regions have different effects on MCI. Therefore, we separate brain nodes with different correlations by maximizing the variance of the brain node attention map. At the same time, the high value of highly correlated brain regions in the brain node attention map will lead to excessive attention loss. In this regard, we control attention loss by minimizing their mean values as follows:

(9) $$\begin{array}{r} L_{\text{att}}=\overset{n}{\underset{x=1}{\sum }}\mathrm{mean}\left(\text{Z}\left(x\right)\right)-\mathrm{var}\left(\text{Z}\left(x\right)\right) \end{array}$$

where $L_{\text{att}}$ denotes the attention loss, mean(·) denotes the mean operation, and var(·) denotes the variance operation.

### 3.4. Spatiotemporal Feature Fusion

In order to further explore the impact of temporal and spatial features of functional signals on MCI identification, we fuse spatial and temporal features. Specifically, we realize the spatiotemporal feature fusion by summing temporal features $f_{j}^{l+1}$ and spatial features ${\hat{f}}_{j}^{l+1}$:

(10) $$\begin{array}{r} h_{j}^{l+1}=f_{j}^{l+1}+{\hat{f}}_{j}^{l+1} \end{array}$$

where $h_{j}^{l+1}\in {\mathbb{R}}^{n\times \frac{\text{T}-w+1}{s}\times c_{2}}$ denotes the output of spatiotemporal features fusion.

Finally, in the *L*-th convolutional layer, we further extract spatiotemporal features by convolution operations and compress them into a scalar as the input of the fully connected layer as follows:

(11) $$\begin{array}{r} S=u_{j}^{L}*h_{j}^{L} \end{array}$$

where $S\in {\mathbb{R}}^{1\times 1\times c_{2}}$ denotes the output of the convolution operation, $u_{j}^{L}$ denotes the convolution kernel set corresponding to $h_{j}^{L}$.

### 3.5. Objective Function

In DSTAN, the features of functional signals are mapped to the corresponding label space through fully connected layers. In the training process, the objective function of the DSTAN network is designed:

(12) $$\begin{array}{r} L_{\text{total}}=\overset{N}{\underset{h=1}{\sum }}L_{ce}\left(f{\left(x,t\right)}_{h},y_{h}\right)+L_{att_{h}}\left(x\right) \end{array}$$

where $L_{ce}\left(\cdot \right)$ denotes the cross-entropy loss, and $L_{att_{h}}$ denotes the attention loss of the *h*-th sample.

## 4. Experiments

In the MCI identification experiments, we utilize fMRI data to train a deep neural network framework for MCI identification. Since the framework needs to use functional connections between brain nodes to extract the spatial features of functional brain signals. Therefore, we use the Pearson correlation coefficient method to construct functional brain networks to obtain functional connectivity matrix related to brain nodes.

### 4.1. Experimental Setting

In this section, our experimental setup is divided into the following steps:

Step 1: In order to obtain the connection matrix of brain nodes, we first use the Pearson coefficient to measure the correlation between brain nodes, so as to obtain a functional connectivity matrix **P**. Then, we sparse the connectivity matrix, where λ=0.1,0.2,…,1 denotes sparsity. Finally, the spatial features of a functional signals are extracted by using graph convolution operation of auxiliary of the connectivity matrix.

Step 2: We set the following settings for each module in DSTAN: 1) In the ST-CONV module, we use convolution kernels to extract time series features, and at the same time, we perform an average pooling operation on the time series. The number of output channels is set to (8,16,32). 2) In the Node-ATT module, we perform operational down-sampling on the base layers and set the sampling rate to 1/16.

### 4.2. Implementation

All experiments are programmed and implemented as follows: PyTorch 1.9 framework, Python version 3.8, and trained with one GeForce RTX 3090 GPU. We use SGD as the optimizer for training, with the momentum of 0.1, weight attenuation of 1e-4, 90 iterations, the initial learning rate of 0.1, attenuation of 50% every 30 times, and batch size of 32. Note that we randomly divided the preprocessed fMRI data obtained in Section 2.2 into a training set and a test set in a ratio of 8:2 for the following experiments.

### 4.3. Evaluation Standard

We use the following indicators for quantitative measurements, which include accuracy, sensitivity, and specificity. All methods are tested with these metrics, which are as follows:

(13) $$\begin{array}{r} \text{Accuracy}=\frac{\text{TruePositive}+\text{TrueNegative}}{\text{TruePositive}+\text{FalsePositive}+\text{TrueNegative}+\text{FalseNegative}} \end{array}$$

(14) $$\begin{array}{r} \text{Sensitivity}=\frac{\text{TruePositive}}{\text{TruePositive}+\text{FalseNegative}} \end{array}$$

(15) $$\begin{array}{r} \text{Specificity}=\frac{\text{TrueNegative}}{\text{TrueNegative}+\text{FalsePositive}} \end{array}$$

where TruePositive represents the number of correctly classified positive patients with MCI, and TrueNegative, FalsePositive, and FalseNegative represent the corresponding number of subjects, respectively.

### 4.4. Experimental Results and Analysis

#### 4.4.1. Visualization of Brain Node Functional Connectivity Matrix

**Figure 4:**
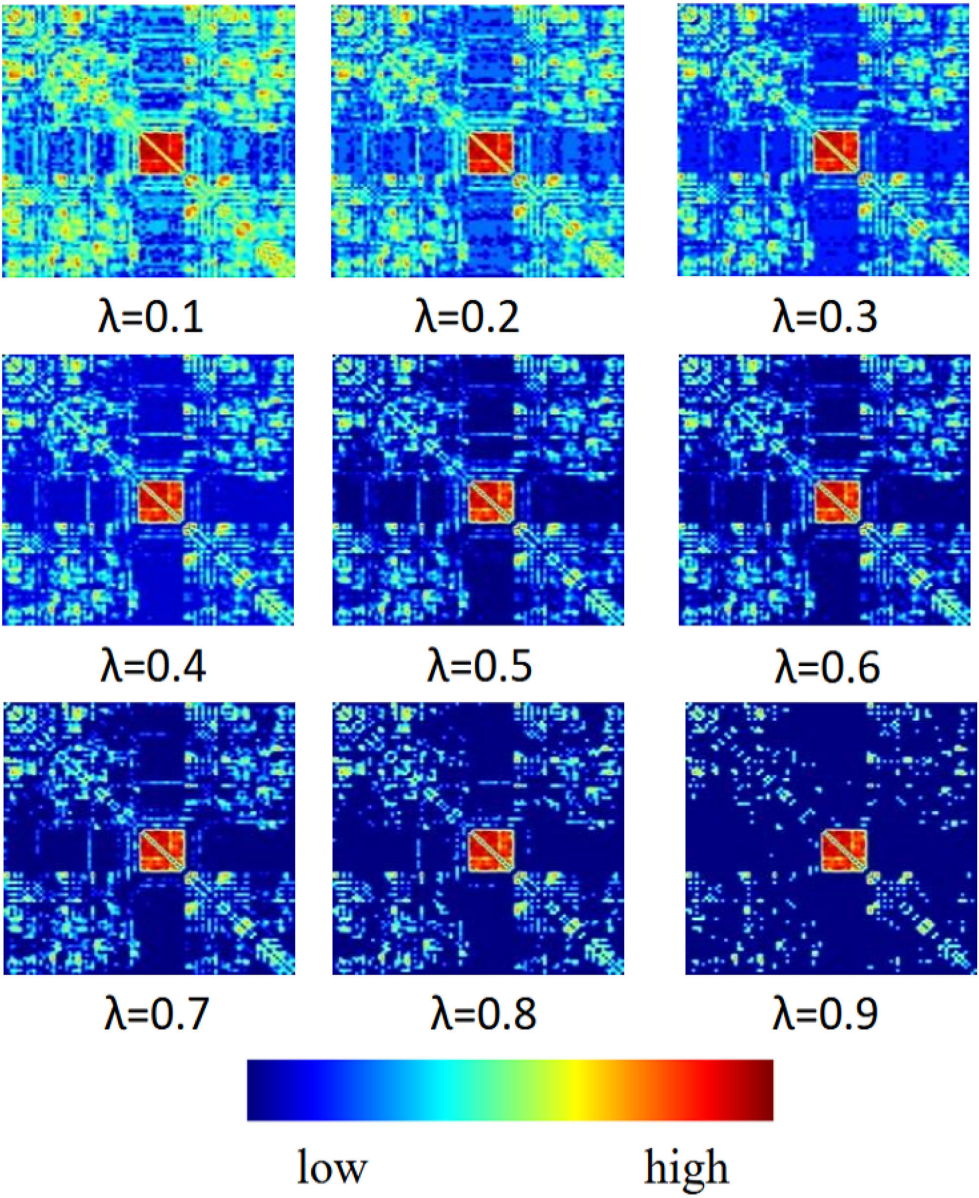
Visual illustration of brain node connections.

In this section, we report the influence of sparsity λ and functional connectivity between brain nodes on MCI identification. We sparse the functional connectivity matrix **P** to different degrees. From Figure 4, we can observe that: 1) in the first row of images, when λ = 0.1, the connectivity matrix **P** retains more brain node connections with weaker correlations, which makes it difficult to extract effective spatial features from functional signals, thus negatively affecting MCI identification. 2) In the middle row of images, when λ = 0.5, the connectivity matrix **P** removes the connections of weakly correlated brain nodes and retains certain correlated brain nodes, which reduces the adverse factors for identifying MCI. 3) In the last row of images, when λ = 0.9, the connectivity matrix **P** retains the highly correlated brain node connections so that the graph convolution operation can extract more effective spatial features, which can further promote the accuracy of model recognition MCI. The above experimental results show that the choice of sparsity λ has a significant dependence on the functional connections between brain nodes, and a higher sparsity has a beneficial impact on spatial feature extraction and MCI identification.

#### 4.4.2. Classification Performance of Different Sparsity

**Figure 5:**
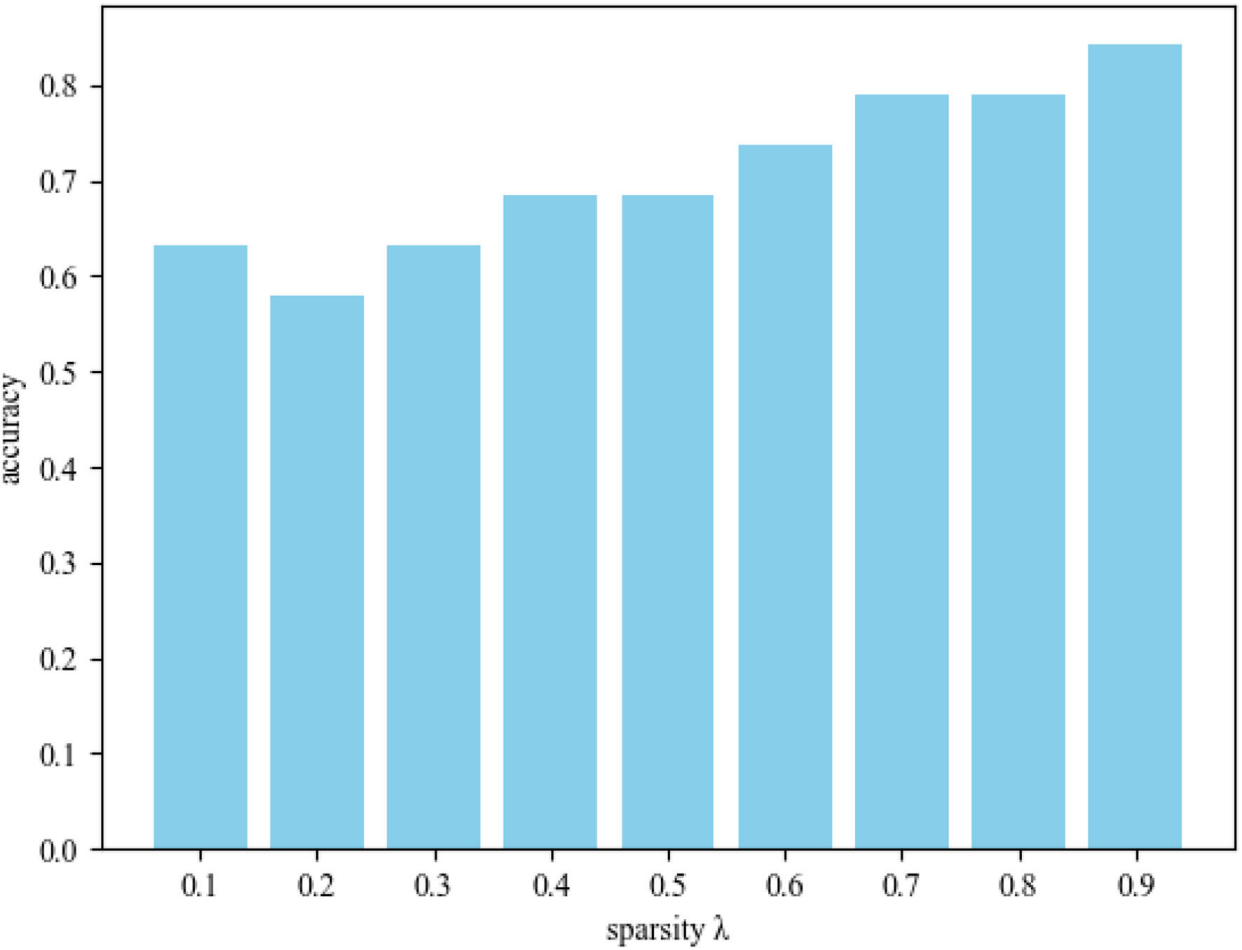
Classification accuracy of different sparsity λ.

In order to further explore the influence of sparsity λ on MCI identification, we conducted experiments in different sparsity ranges. Figure 5 shows the sparsity λ classification accuracy histogram in the range of 0.1–0.9. From this figure, we obtain the following observations: 1) When λ = 0.9, DSTAN classification accuracy is significantly better than other sparse classification experiments. 2) The functional connections of brain nodes affect the classification performance of MCI, which leads to great differences in the classification results of connection matrices with different sparsity. 3) With the increase of sparsity, the interference of weakly correlated brain nodes gradually decreases, and the classification accuracy improves. Therefore, removing weak functional connections between brain nodes in DSTAN can improve MCI identification performance. Finally, the above experimental results prove again that higher sparsity can promote graph convolution to capture more spatial features and further improve MCI classification accuracy.

#### 4.4.3. MCI Identification

We performed MCI vs. NC experiments on the MCI dataset. We compare the following methods, including traditional machine learning methods: Support Vector Machine (Song et al., 2017), RandomForest (Fredo et al., 2018), and Deep learning methods: Multi-Layer Perception (Shanmuganathan, 2016; Almuqhim and Saeed, 2021; Gao et al., 2021; Yin et al., 2021). Table 2 reports the test accuracy of all methods on the MCI dataset. The following observations are made: 1) In MCI identification, deep learning methods are significantly better than traditional machine learning methods. 2) The DSTAN method significantly outperformed other methods in accuracy, sensitivity, and specificity. In addition, this method is effective for MCI identification based on FBN. In conclusion, DSTAN can well identify patients with MCI, and the probability of misdiagnosis of patients with NC is low.

**Table 2:** Performance of all methods on MCI identification.

**Method**	**Accuracy**	**Sensitivity**	**Specificity**
SVM	63.15	75.00	54.54
RF	68.42	54.54	75.00
MLP	68.42	75.00	63.63
Gao	78.94	72.72	**87.50**
Almuqhim	63.15	63.63	**87.50**
YIN	73.68	**81.81**	62.50
DSTAN	**84.21**	**81.81**	**87.50**

*The optimal performances are bolded*.

#### 4.4.4. Visualization of Brain Node Attention Map

**Figure 6:**
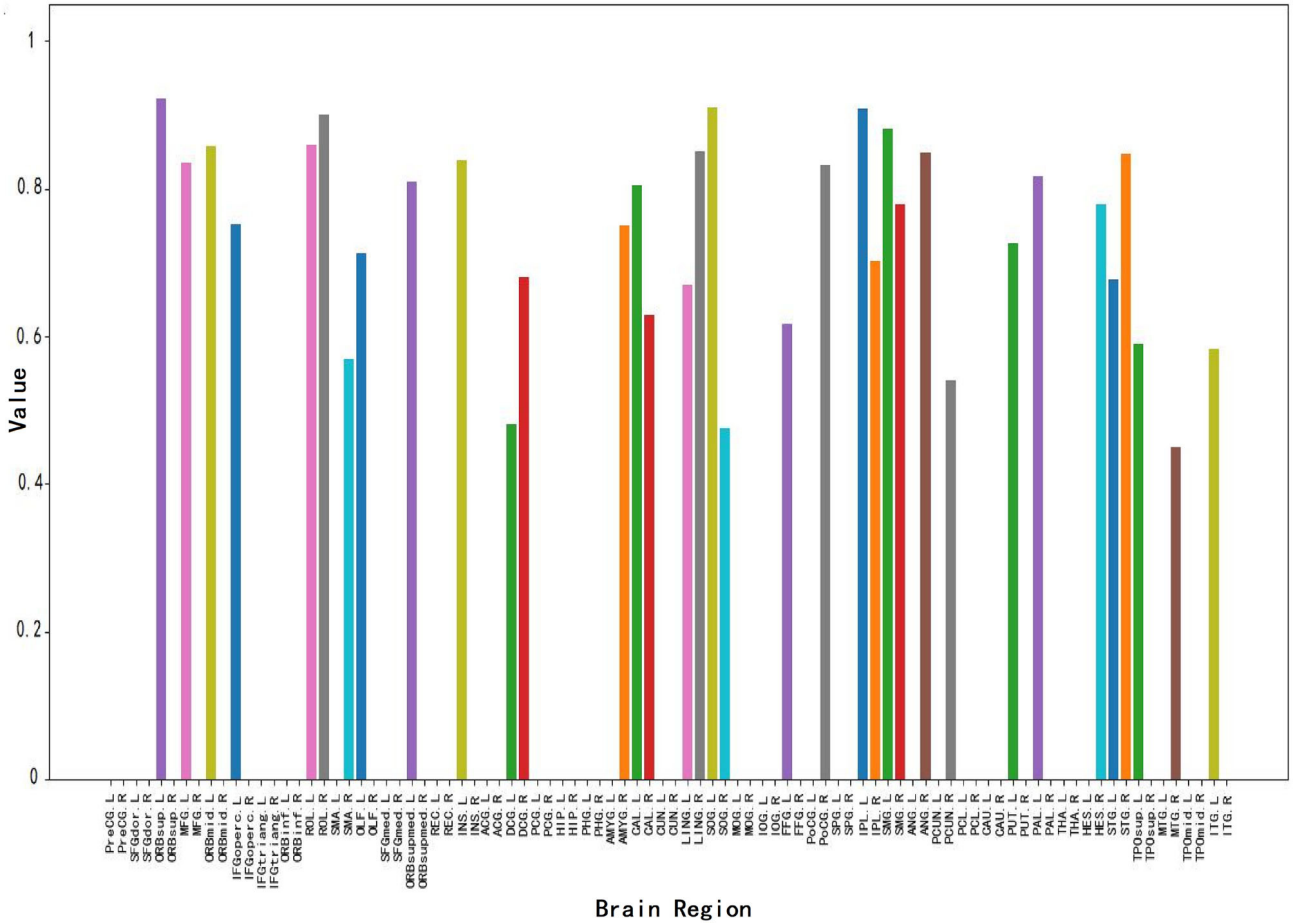
Visualization of brain node attention map.

Figure 6 shows the brain node visualization obtained from the attention map in Section 3.3. Specifically, the abscissa values correspond to the brain regions of different brain nodes, and the ordinate values represent the correlation intensity. The higher the value of the ordinate, the stronger the correlation between the corresponding brain region and MCI. The colors of corresponding values in all brain regions are randomly generated. From this figure, we can find: 1) The corresponding values of most brain regions are 0, i.e., Inferior frontal gyrus, triangular part (IFGtriang), and Gyrus rectus Middle (REC) occipital gyrus (MOG). This result indicates that this part of the brain region has nothing to do with MCI identification, which is consistent with the conclusion of Wee et al. (2012) on the relationship between brain regions and MCI. 2) This figure shows a total of 34 brain regions that have a strong correlation with MCI, thus affecting MCI identification. 3) This figure shows that brain regions such as the middle temporal gyrus (MTG), Superior frontal gyrus medial orbital (ORBsupmed), inferior parietal (IPL), Supramarginal gyrus (SMG), and Precuneus (PCUN) have a strong correlation with MCI identification which is consistent with previous MCI imaging biomarker reports and pathological studies (Greicius, 2008; Albert et al., 2011).

### 4.5. Ablation Studies

To verify the effectiveness of each component in DSTAN proposed in this article, we perform ablation studies. Table 3 reports the performance comparison between DSTAN and the removal of the attention mechanism (No-Att for short). From this table, we observe that: 1) In the No-Att method, the gap between DSTAN and No-Att accuracy is small. But the sensitivity and specificity are much lower than DSTAN. This finding may suggest that we are more likely to misdiagnose patients with MCI and patients with misdiagnosed NC. 2) DSTAN is superior to the No-Att method in all evaluation indicators. The above results demonstrate that the attention mechanism in the DSTAN framework is used to eliminate the interference of redundant brain nodes on MCI identification, so as to improve the performance of MCI classification.

**Table 3:** Comparing the classification performance of the DSTAN and the No-Att methods.

**Method**	**Accuracy**	**Sensitivity**	**Specificity**
No-Att	81.81	62.50	73.68
DSTAN	**84.21**	**81.81**	**87.50**

*Performance of all methods on MCI identification. The optimal performances are bolded*.

## 5. Discussion

In this study, a reliable functional brain network (FBN) is constructed from functional magnetic resonance imaging (fMRI) data to assist in the identification of mild cognitive impairment. Different from previous studies, we propose a novel DSATN framework, which fuses functional brain signals and spatiotemporal features of FBN for MCI identification. Specifically, we first capture spatiotemporal features through ST-CONV strategy and graph convolution. Then, we capture the brain node features associated with MCI through an attention mechanism. Finally, we fuse these features for DSATN network training. Our detailed experimental results are listed as follows: 1) We facilitate graph convolution to obtain more effective spatial features in functional brain signals through a higher sparse functional connectivity matrix. 2) We use the attention mechanism to effectively improve the MCI identification performance and capture 34 brain regions with strong correlations with MCI. 3) We obtain an encouraging classification accuracy of 84.21% on MIC identification.

### 5.1. Spatiotemporal Feature Fusion in MCI Identification

Functional magnetic resonance imaging (fMRI) is a widely used neuroimaging modality. This modality performs imaging by measuring the blood oxygen level dependence (BOLD) of each brain region in the brain (Khosla et al., 2019). fMRI data are rich in temporal and spatial features (Ma et al., 2016). Previous study has studied the spatial features of fMRI, e.g., using matrix decomposition (Du and Zhang, 2021), Pearson correlation sparse (Smith et al., 2013), and sparse representation (Lee et al., 2011) to construct FBN, and extract its structural features; At the same time, there are also studies on the temporal features of fMRI, e.g., using Rnn (Dvornek et al., 2018), LSTM (Yan et al., 2018) to extract temporal features in the time series of fMRI data. Some recent studies have investigated the spatiotemporal features of fMRI data, e.g., in Gadgil et al. (2020), the authors divided the fMRI data into multiple short sequences according to the length of the time series, then quantified the connectivity between brain regions in the short sequences, and used graph convolution to extract spatial features of short sequences. In Li et al. (2020a), authors used convolution operation to extract spatial features in fMRI data, and taked the resulting features as the input of LSTM network to capture the temporal information contained in the data. The above methods utilize spatiotemporal features in fMRI data, but do not deeply consider the relationship between temporal and spatial features. In DSTAN, considering that both temporal and spatial features of fMRI data have positive effects on MCI identification, we further fuse temporal and spatial features. Specifically, we use convolution operation to extract temporal features and graph convolution operation to extract spatial features. Then, we achieve spatiotemporal feature fusion by element-wise summation. Extensive experimental results are compared with current state-of-the-art methods to verify the effectiveness of spatiotemporal feature fusion. We speculate as follows: 1) Each brain region corresponds to a set of time series and contains temporal information. 2) The corresponding temporal features of the brain regions related to MCI have a positive role in promoting MCI identification, and their corresponding spatial features have a key role in MCI identification. Therefore, the accumulation of these two positive-acting features can improve the performance of MCI identification.

### 5.2. Brain Node Attention Mechanism in MCI Identification

In the brain node attention module, we set up multiple base layers to capture the brain regions related to MCI. The experiments in this article found that 34 brain regions in all brain regions are closely related to MCI, including the middle temporal gyrus controls semantic cognition (Davey et al., 2016); the Superior frontal gyrus medial orbitally affects schizophrenia and delusions (Gao et al., 2015); Inferior parietal affects sensory memory function Chen et al. (2021); Supramarginal gyrus affects auditory memory function (DES, 2014); and Precuneus affects cognitive function (Nagano-Saito et al., 2021). These brain nodes are correlated with MCI and are consistent with the experimental results of previous studies (Greicius, 2008; Albert et al., 2011). At present, many studies have shown that FBN can show more structures or attributes, such as classification, hierarchy, centrality, synchronization, and scale-free topological results. Therefore, we will further explore the relationship between brain regions and MCI, and use correlation knowledge sharing in multi-task learning for MCI identification and interpretability research, providing a new method for the prevention and treatment of MCI.

### 5.3. Limitations and Future Directions

We build an MCI identification mechanism based on spatiotemporal feature fusion and attention mechanism and achieve excellent experimental results. However, there are still several limitations that need to be considered further. First, the training and validating model is inseparable from a large number of data samples and data from different sources. In future study, we need to further validate the robustness of our proposed method with large samples and heterogeneous data from multiple sources. Second, less research on the interpretability of MCI identification is involved. We need an interpretable analysis combined with clinical knowledge. Third, MCI is an early stage of AD, and MCI should be analyzed together with other related nervous system diseases. At present, many studies have shown that FBN can show more structures or attributes, such as classification, hierarchy, centrality, synchronization, and scale-free topological results. Therefore, we will further explore the relationship between brain regions and MCI, and use correlation knowledge sharing in multi-task learning for MCI identification and interpretability research, providing a new method for the prevention and treatment of MCI.

## 6. Conclusion

In the present study, we propose a DSTAN network, which uses spatiotemporal feature fusion and attention mechanism for MCI identification, and obtains excellent classification performance (Accuracy = 84.21%). In addition, spatiotemporal feature fusion increases the diversity of effective training samples by accumulating temporal and spatial features. The brain node attention mechanism strengthens the model's attention to brain regions related to MCI. Our findings demonstrate that the combined use of spatiotemporal feature fusion and attention mechanism can better distinguish MCI from NC. Combining FBN and graph convolution for better MCI identification is helpful for early clinical diagnosis of AD.

## Data Availability Statement

Publicly available datasets were analyzed in this study. This data can be found here: https://www.nitrc.org/projects/modularbrain/.

## Ethics Statement

The studies involving human participants were reviewed and approved by Department of PET/MR, Universal Medical Imaging Diagnostic Center, Shanghai, China. The patients/participants provided their written informed consent to participate in this study.

## Author Contributions

QF and YH designed this experiments and wrote the manuscript. YL drawed and wrote the manuscript. LG was involved in funding acquisition and wrote the manuscript. XG provided and processed data. All authors contributed to the article and approved the submitted version.

## Funding

This project is supported by Wuyi University- Hong Kong- Macau Joint Fund: 2019WGALH23, Teaching Reform Project of Guangdong Province: GDJX2020009, Scientific Research Subjects of Shanghai Universal Medical Imaging Technology Limited Company: UV2020Z02 and UV2021Z01, and Shanghai Municipal Commission of Health and Family Planning Science and Research Subjects: 202140464.

## Conflict of Interest

YL was employed by Nanjing Huayin Medical Laboratory Co., Ltd. The remaining authors declare that the research was conducted in the absence of any commercial or financial relationships that could be construed as a potential conflict of interest.

## Publisher's Note

All claims expressed in this article are solely those of the authors and do not necessarily represent those of their affiliated organizations, or those of the publisher, the editors and the reviewers. Any product that may be evaluated in this article, or claim that may be made by its manufacturer, is not guaranteed or endorsed by the publisher.
